# Where I am from matters: factors influencing behavioral and emotional changes in autistic individuals during COVID-19 in Latin America

**DOI:** 10.3389/fpsyt.2023.1283326

**Published:** 2023-12-22

**Authors:** María Cecilia Montenegro, Ana C. Ramírez, Juventino Hernandez Rodriguez, Bianca T. Villalobos, Gabriela Garrido, Cecilia Amigo, Daniel Valdez, Natalia Barrios, Sebastián Cukier, Alexia Rattazzi, Analía Rosoli, Ricardo García, Cristiane S. Paula, Georgina Pérez Liz, Cecilia Montiel-Nava

**Affiliations:** ^1^Department of Psychological Science, University of Texas Rio Grande Valley, Edinburg, TX, United States; ^2^Universidad de la República, Montevideo, Uruguay; ^3^Facultad Latinoamericana de Ciencias Sociales (FLACSO), Ayacucho, Argentina; ^4^Programa Argentino para Niños, Adolescentes y Adultos con Condiciones del Espectro Autista (PANAACEA), Buenos Aires, Argentina; ^5^Organización Estados Iberoamericanos para la Educación, la Ciencia y la Cultura (OEI), Santo Domingo, Dominican Republic; ^6^Universidad de Chile, Santiago, Chile; ^7^Developmental Disorder Program, Mackenzie Presbyterian University, São Paulo, Brazil; ^8^A.J. Drexel Autism Institute, Drexel University, Philadelphia, PA, United States

**Keywords:** autism spectrum disorder, COVID-19, dysregulated behaviors, age, gender, coexistence

## Abstract

**Background:**

The COVID-19 pandemic brought an increased incidence of disease and mortality in the world at large, making it a particularly salient and stressful life event. For those individuals residing in Latin America, the pandemic was met with fragmented healthcare systems, economic downturn, and sociopolitical crisis which puts autistic individuals at risk for more detrimental outcomes. Behavioral and emotional challenges experienced by autistic individuals at the beginning of the pandemic could later develop into more severe symptomatology as the pandemic progresses. The present study aimed to explore changes in dysregulated (overt and internalizing) behaviors and preoccupation with getting sick during the COVID-19 pandemic among autistic children in 7 Latin American countries.

**Method:**

Sample consisted of 1,743 caregivers, residing in: Argentina (*n* = 677, 38.8%) Brazil (*n* = 156, 9%), Chile (*n* = 251, 14.4%), Dominican Republic (*n* = 171, 9.8%), Mexico (*n* = 126, 7.2%), Uruguay (*n* = 259, 14.9%) and Venezuela (*n* = 103, 5.9%). The majority of caregivers who completed the questionnaire were mothers (85.1%), and most had a male autistic child (81.6%). A series of independent sample *t*-tests were conducted to assess country differences in dysregulated behaviors and preoccupation with getting sick. Linear regressions were conducted to identify which demographic characteristics and micro-level contextual factors predicted dysregulated overt behaviors and psychological changes.

**Results:**

Contextual factors, such as country of residence, were related to preoccupation with getting sick and dysregulated behavior. Particularly, residing in Mexico and Brazil were related to changes in preoccupation with getting sick and mental health concerns. Coexistence predicted dysregulated internalizing behaviors, while being older significantly predicted preoccupation with getting sick. Increased screen time only predicted anxiety.

**Conclusion:**

Our findings highlight differences and predictions of behavioral challenges and psychological changes based on certain contextual factors and individual characteristics while experiencing severe life stressors such as a worldwide pandemic. This knowledge could help inform policies and decrees aimed at protecting those most vulnerable due to their increased difficulty adapting to change.

## Introduction

In 2020, the world drastically changed as a result of the COVID-19 pandemic (1). Latin America (LATAM) was particularly impacted by the pandemic with one-third of all COVID-19 cases in the world and 34% of COVID-19 related deaths at the start of the pandemic (2, 3). This rate is expected to be higher than reported since COVID-19 testing in LATAM was insufficient due to limited resources and poor capacity (4, 5).

The known high rate of COVID-19 cases and deaths was likely perpetuated by a large treatment gap, poor health infrastructure, economic inequality, and political instability (6–10). In LATAM, most countries have fewer hospital beds and health professionals *per capita* than countries in more developed regions and thus an increased difficulty in meeting an increased demand (9, 10). Also, due to the newly implemented containment measures, in which many healthcare services were transitioned to online platforms, health professionals were met with the added challenge of modifying service delivery to electronic modalities but limited resources to do so (6). Financially COVID-19 came at a time when the region’s Gross Domestic Product (GDP) had been declining in a way not experienced since 1950 (10).

Sociodemographic factors such as infrastructure, financial and sociopolitical environment can modify the impact of the pandemic. Moreover, throughout the pandemic, existing social discontent translated to lower trust in their government’s handling of the pandemic (11). In LATAM, this lack of trust, in addition to aid shortages and an inability to afford isolation, meant decreased desire to comply with containment measures and thus increased probability of getting sick and infecting loved one.

When assessing the impact of COVID-19 in terms of psychological distress, a study by Silverio-Murillo et al. identified increased anxiety, insomnia and stress-related Google searches at the time when countries began employing stay-at-home orders (12). These results were further supported by a systematic review by Zhang et al., which assessed the prevalence of mental health symptoms in LATAM (13). Their findings showed high rates of depression and anxiety, yet it observed higher insomnia prevalence compared to Europe and Asia. This result could be due to poorly executed safety protocols and non-inclusive policies implemented after the pandemic (14, 15), thus increasing distress, particularly among historically vulnerable populations, such as autistic individuals in LATAM countries (16, 17).

### COVID-19 and autism spectrum disorder

About one in every hundred children is diagnosed with autism spectrum disorder worldwide (ASD; (18)). In Latin America, prevalence rate is difficult to estipulate due to the limited number of epidemiological studies available (19). Of the few countries with known prevalence rates, numbers range from 17 to 52.6 in every in every 10, 000 individuals in Venezuela and Argentina, respectively, (20, 21). ASD is characterized by difficulties with social communication, repetitive behavior patterns, and focused interests (22). Also, some autistic individuals might experience difficulties shifting focus making it more challenging to distract themselves from a stressful life event (8). This impairment in cognitive flexibility is problematic when we consider that shifting one’s perspective could help in the recovery process (8). Additionally, autistic individuals typically adhere to strict routines, and the disruption of these routines can be met with high levels of distress (23). Because of these characteristics, it is possible that autistic individuals perceive stressful life events more distressing or traumatic than non-autistic individuals (24). In the context of COVID-19 in which routines changed quickly (e.g., interruption of face-to-face schooling and therapeutic services and limitation of leisure activities), autistic individuals might have experienced it as a significant disruption to their lives (25) which could explain reports of increased fear, uncertainty, psychological distress, and psychosomatic difficulties at the start of the pandemic (25, 26).

### Contextual micro and macro factors

The present study focuses on the influence of particular micro and macro environmental factors on dysregulated behaviors and psychological differences among autistic individuals throughout the start of the pandemic in LATAM (27, 28). At the micro-level, research indicates that household overcrowding predicted COVID-19 infection rates and mortality (29, 30). Furthermore, household overcrowding has been associated with worsening of behavioral problems in children (31). Moreover, housing quality and overcrowding can negatively impact household dynamics, family wellbeing, and overall health outcomes (32). Poor housing conditions and overcrowding can further complicate the increased need for isolation and quarantine to mitigate the surge of COVID-19 cases (33). The pandemic also shed light on other contextual issues. Due to limited social outings, individuals screen time usage increased, thus, negatively impacting overall emotional and physical health (34–37). This increase could be particularly taxing, considering that previous literature shows that increased screen time was related to increased irritability, concentration difficulties, mood disturbances, anxiety, and sleep dysregulation (36). This is especially relevant in the case of autistic individuals given the already high prevalence of co-occurring sleeping problems (38, 39).

At the macro level, COVID-19 related deaths and healthcare infrastructure could play a role in the functioning of autistic individuals. Research has indicated that in those countries harder hit by the pandemic there was an increase in mental health conditions (40). In LATAM autistic individuals faced particular circumstances as the increased rate of severe COVID-19 meant an increased demand for healthcare infrastructure that was already fraught in most countries (16, 41). Argentina, a country in our sample, had the largest number of available hospital beds per 1,000 individuals in LATAM (42); however, there were fewer than in other higher-income countries (e.g., Japan) (43). Before the pandemic started, the WHO used an overall efficiency index (OEI) to measure health system performance. This composite index assesses the efficiency of health systems in meeting specific goals, such as responsiveness (e.g., distribution), adequate financing, and inequality (44). According to the WHO, Chile has the highest OEI in Latin America, whereas Brazil scores almost half of it (45). However, Chile’s healthcare expenditure falls well behind other countries with similar OEI such as Australia and Canada exposing some of the healthcare challenges in the region (44, 45).

#### Dysregulated behaviors and preoccupation with getting sick

Bauminger et al. described a cluster of ASD behaviors that predict social difficulties and peer unacceptance (46). These behaviors can be divided into two categories, dysregulated overt (externalizing) and internalizing behaviors. According to this classification, dysregulated overt behaviors include aggression, impulsivity, and impulse-control problems, while dysregulated internalizing behaviors include social withdrawal, anxiety, and mood issues (46). Past research has shown that the phenotypic expression of these dysregulated behaviors might vary depending on the gender of the autistic person, with females being more likely to experience internalizing behaviors, whereas males are more likely to exhibit overt behaviors (47, 48).

Life stressors, contextual factors, and individual characteristics can intensify dysregulated behaviors among autistic individuals (49–52). Given the significant changes and disruptions as a result of the COVID-19 outbreak, the pandemic has heightened levels of distress in autistic individuals, potentially leading to behavioral and emotional disturbances, as well as emotional duress (51, 53). Although multiple studies have explored the pandemic’s impact on autistic individuals, most of these have been conducted in developed and high-income countries in Europe and North America. Amorim et al. compared behavioral changes in neurotypical and autistic children during the COVID-19 pandemic in Portugal (49). Their findings suggested that autistic individuals experienced behavioral changes (e.g., irritability, obsessiveness, hostility, impulsivity) and increased anxiety when compared to their neurotypical peers. Also, more than half of the autistic individuals in their sample experienced difficulties with emotional regulation (49). Furthermore, caregivers of autistic individuals identified social isolation, limited opportunity for outdoor activities, boredom, and increased online activity as the most problematic challenges experienced by their children during the pandemic (49).

Given the dearth of information regarding the impact of COVID-19 in autistic individuals in LATAM, the present study had three aims. First, this study sought to understand micro and macro contextual influences (e.g., household overcrowding and screen time) on behavioral and psychological changes in autistic individuals, as perceived by their caregivers, in LATAM as a result of COVID-19. Specifically, the study assessed differences in preoccupation with getting sick and overt and internalizing behaviors of autistic individuals, depending on their country of residence, COVID-19 related deaths, hospital beds per 1,000 individuals, and OEI. Second, the study examined micro-level contextual factors as predictors of dysregulated behaviors among autistic individuals as perceived by their caregivers. Lastly, the study assessed autistic individuals’ demographics (e.g., age and gender) and their relationship to preoccupation with getting sick and overt and internalizing behaviors.

## Methods

### Sample

The sample consisted of 1,743 caregivers residing in 7 LATAM: Argentina (*n* = 677, 38.8%), Brazil (*n* = 156, 9%), Chile (*n* = 251, 14.4%), Dominican Republic (*n* = 171, 9.8%), Mexico (*n* = 126, 7.2%), Uruguay (*n* = 259, 14.9%), and Venezuela (*n* = 103, 5.9%). Data collection occurred from May 2020 to August 2020. The majority of participants were female (85.1%) and had a male autistic child (81.6%, see Table 1). To be included in the study, caregivers needed to be least 18 years old and have an autistic child, and be a resident of the included LATAM countries at the time of the study. Recruitment occurred through community support groups for caregivers with autistic youth, service agencies, physicians’ offices, social media groups, and word of mouth.

**Table 1:** Demographic characteristics of the sample (*N* = 1,743).

	*N*	%
*Country*		
Argentina	677	(38.8)
Brazil	156	(9.0)
Chile	251	(14.4)
Dominican Republic	171	(9.8)
Mexico	126	(7.2)
Uruguay	259	(14.9)
Venezuela	103	(5.9)
*Type of Caregiver*		
Father	171	(9.8)
Mother	1,484	(85.1)
Grandparent	23	(1.3)
Other	65	(3.8)
*Diagnosis of Child*		
Autism	295	(17.5)
Asperger	240	(14.3)
Pervasive Developmental Disorder (PDD)	144	(8.5)
PDD-Not Otherwise Specified	135	(8.0)
ASD	870	(51.7)
*Gender of Child*		
Male	1,423	(81.6)
Female	320	(18.4)
*Age of Autistic Child*		
0–6 years old	622	(35.7)
7–12 years old	673	(38.6)
13–18 years old	294	(16.9)
19+ years old	154	(8.8)

### Procedure

The present study is part of a larger research project conceptualized by a group of researchers and professionals who in 2015 formed an alliance called the *Red Espectro Autista Latinoamérica* (REAL) to help deepen the understanding of ASD in Latin America. These researchers developed this network as a response to a call made by previous publications to fill the ASD knowledge gap in other parts of the world outside the United States and Europe (7, 54). At the pandemic’s start, REAL clinicians and researchers observed many rapid changes (e.g., lockdown, changes in service modalities, safety measures (6) and were interested in understanding the experience of autistic individuals and their families).

For this study, participants completed an online survey (google forms), which inquired about different aspects of the autistic individual sociodemographic characteristics, as well as service utilization in each of the included countries. Recruitment was conducted through community support groups, service agencies, pediatrician offices, word of mouth, and social media groups. Study procedures were approved by the Institutional Review Board (IRB) of The University of Texas Rio Grande Valley and the ethics boards of the different agencies with which the other national coordinators were affiliated. Data collection occurred from May 2020 until August 2020. Data was filled out in excel files in each country and merged in SPSS by one of the main researchers and two of her research assistants.

#### Instrument

The REAL network developed an online survey to inquire about the impact of the COVID-19 pandemic on autistic individuals in LATAM. Due to the novelty of the COVID-19 pandemic and the lack of instruments with psychometric properties normed with the population included in this study, a new instrument was developed by professionals (e.g., psychiatrists, psychologists, doctoral students, and researchers) from the countries included. The development of this survey was comprised of six stages: (1) meetings among professionals from each LATAM country to discuss needs being observed in autistic individuals; (2) drafting of questions to be included in the survey; (3) consensus for relevance and appropriateness of each item; (4) pilot testing to select the final items, and (6) pilot testing the final draft of the survey to assess its acceptability and relevance among families. The instrument was designed in Spanish and pilot tested to ensure its appropriateness for cross-cultural use. The principal researcher from Brazil and a Ph.D. student translated the questionnaire to Portuguese for the Brazilian sample. Throughout a series of meetings, agreement of the translated instrument was achieved. This translated document was also pilot tested with two mothers of autistic individuals to ensure questions were understandable, relevant, and non-stigmatizing.

### Statistical analyses

For exploring countries’ differences in preoccupation with getting sick and dysregulated behaviors of autistic individuals depending on country’s infrastructure, health services efficiency and COVID-19 related deaths, independent sample *t*-tests were conducted (Tables 2, 3). Before the analyses were conducted, assumption of normality was assessed and range of skewness (SE < 3) and kurtosis (<10) were utilized (57). Both preoccupation with getting sick and dysregulated behaviors were normally distributed. For homogeneity of variance, the assumption was met as assessed with *F*_max_ with a maximum value of 10 accepted (58). The rest of the study’s aims were tested by five linear regression analyses conducted separately. These attempted to (1) predict dysregulated overt behavior among those who reside in more crowded conditions (more habitants living in the same household, fewer squared meters, and no yard) and those who reported worsening coexistence (Table 4); (2) predict anxiety, preoccupation with getting sick, and mood among those who increased screen time use (Table 5); (3) predict dysregulated overt and internalizing externalizing behaviors among different gender (Table 6); and (4) predict if older autistic individuals experienced increased preoccupation with getting sick (Table 7). To test assumptions before conducting these linear regressions, independence of residuals were assessed by the Durbin-Watson statistic, and only values between one and three were accepted (59). To ensure linear relationship with dependent variables, independent variables were dummy coded (58). Finally, homogeneity of variances was assessed with *F*_max_ ratio. All statistical analyses were conducted using IBM SPSS, version 26.

**Table 2:** COVID-19 cases and deaths, overall efficiency index, and medical capacity.

	COVID-19-cases adjusted by population (%)	COVID-19-deaths adjusted by COVID cases (%)	Hospital Beds per 1,000	Overall Efficiency Index
Country				
Brazil	9.8	2.8	2.1	0.57
Argentina	11.4	2.2	5.0	0.72
Uruguay	11	1.6	2.4	0.75
Mexico	2.6	7.7	1.0	0.76
Venezuela	1.2	1.2	0.9	0.78
Dominican Republic	3.2	1.1	1.6	0.79
Chile	8.5	2.3	2.1	0.87

Data from The World Bank (55) for Hospital beds per 1,000, World Health Organization (56) for COVID-19 cases and COVID-19 deaths, and World Health Organization (45) for Overall Efficiency Index.

**Table 3:** Differences in dysregulated internalizing behaviors and preoccupation with getting sick by country.

Country Comparisons	Dysregulated internalizing behaviors			Preoccupation with getting sick		
	M	SD	*t*	M	SD	*t*
COVID-19 Deaths			3.34*			2.13*
Mexico	6.99	1.61		3.19	0.99	
Dominican Republic	6.31	1.79		2.92	1.12	
Overall Efficiency Index			3.32*			3.49*
Chile	7.065	1.74		3.08	1.09	
Brazil	7.63	1.52		3.44	0.85	
Hospital Beds			0.37			0.45
Argentina	7.20	1.58		3.18	0.74	
Venezuela	7.26	1.72		3.15	0.87	

^*^p < 0.05, ^**^p < 0.01, and ^***^p < 0.001.

**Table 4:** Linear regression analysis for crowded conditions predicting dysregulated overt behaviors.

	B	SE	*β*	*T*	95% CI
Crowded condition	9.72	0.27		36.27	9.19–10.24***
Coexistence					
Worst	1.92	0.11	0.30	17.32	1.71–2.14***
People in household					
More than 3	−0.14	0.19	−0.02	−0.72	−0.53–0.25
House size					
More than 50 m^2^	−0.12	0.12	−0.02	−1.04	−0.36–0.11
Outside space					
No	0.17	0.13	0.03	1.32	−0.08–0.43

*R*^2^ = 15.2%. *p* < 0.05, ^**^p < 0.01, and ^***^p < 0.001.

**Table 5:** Linear regression analysis for prediction of anxiety, mood, and sleep dysregulation based on screentime increase.

	Anxiety			Mood			Sleep dysregulation		
	B	*β*	95% CI	B	*β*	95% CI	B	*β*	95% CI
Increased screentime	0.121	0.06	0.02–0.23*	0.09	0.04	−0.01–0.19	0.00	0.05	−0.10–0.10

*R*^2^ = 0.03% for anxiety regression model, *R*^2^ = 0.00 for sleep dysregulation regression model, and *R*^2^ = 0.02 for mood regression model. *p < 0.05, **p < 0.01, and ***p < 0.001.

**Table 6:** Linear regression analysis for gender of autistic child as predictor of dysregulated behaviors.

	Dysregulated internalizing behaviors					Dysregulated overt behaviors				
	B	*β*	*t*	Sig. (*p*)	95% CI	B	*β*	*t*	Sig. (*p*)	95% CI
Gender	0.01	0.002	0.10	0.92	−0.19–0.21	0.05	0.01	0.35	0.72	−0.23–0.34

**Table 7:** Linear regression analysis for age of autistic child as predictor of preoccupation with getting sick.

	B	*β*	*t*	95% CI
Age Group	3.13		129.11	3.09–3.18***
13 and older	0.16	0.08	3.27	0.06–0.25*

*R*^2^ = 0.6%. *p < 0.05, **p < 0.01, and ***p < 0.001.

### Variables

#### Autistic individuals’ characteristics

Caregivers were asked about the age and gender of their child. Response options for age included: 0–6 years old, 7–12 years old, 13–18 years old, and 19 years old and older. For analyses, age was transformed into a binary variable (0 = younger than 12 years of age, 1 = 13 years of age and older) to make comparisons between children and adolescents and young adults. For gender, participants were given three options: female, male, and other.

#### Contextual factors

Caregivers were asked to indicate their country of residence, number of people living in the house (i.e., 2, 3–5, 5+ people living in the household), squared meters of their home, whether they had outside space (e.g., a yard), family coexistence (1-*Much worse than before* to 5-*Much better than before*) and screen time usage (1-*Increased a lot* to 5-*Decreased a lot*). Number of people living in the house was collapsed into a binary variable to better compare crowding (0 = 2 people living in the house, 1 = 3 and more people living in the house). For housing, a house below 50 m^2^ is considered extremely small and characteristic of crowded housing for a family of any size (60, 61), and thus housing size was collapsed into a binary variable (0 = Less than 50 m^2^, 1 = More than 50 m^2^). Lastly, for the comparison of screen time usage, this variable was collapsed into a binary one (0 = No Increased Screen Time, 1 = Increased Screen time). Included in the “No Increased Screen Time” were those that reported a decrease or no change in screen time usage. In the “Increased Screen Time” category, those that reported an increase by “a little” or “a lot” were included. Of the total sample, 80.2% of caregivers reported an increased screen time usage in their children and 19.8% reported no increased screen time.

#### Country differences

Three factors were assessed to predict increased preoccupation with getting sick and dysregulated internalizing behaviors among autistic individuals as reported by their caregivers. First, number of COVID-19 related deaths was used as proxy to assess country’s COVID-19 burden (62, 63). Number of hospital beds per 1,000 people was used as a proxy to evaluate countries’ health infrastructure and resources (55, 64). Lastly, to address overall infrastructure and healthcare system capacity, each country’s OEI was examined (44, 45). Countries with opposing values on the listed factors were compared. In terms of COVID-19 related deaths, Mexico had the most fatalities (7.7%) and the Dominican Republic the least (1.1). When considering infrastructure, Venezuela had the lowest hospital beds per 1,000 people (0.9), whereas Argentina had the most (5). Finally, Brazil had the lowest OEI (0.57), while Chile had the highest (0.87). These countries were compared against each other based on the respective factor of interest (Venezuela against Uruguay, Mexico against the Dominican Republic, and Brazil against Chile).

#### Dysregulated behaviors

The dependent variables consisted of a series of questions asking about behavioral changes experienced by the autistic individual as perceived by their caregiver. Participants responded to these items using a Likert scale, from 1-*Much less than before* to 5-*Much more than before*. An example included: “The person with ASD has been hitting, pinching, biting, or pushing others.” To align with previous literature (46), dysregulated overt behaviors included three items pertaining to irritability, aggression, and concentration; while internalizing behaviors included two items that inquired about changes in anxiety and mood. For dysregulated overt behaviors, Kendall’s tau-*b* correlation there were strong positive associations between concentration difficulty and irritability *τb* = 0.322, *p* < 0.001, concentration difficulty and aggression *τb* = 0.219, *p* < 0.001, and aggression and irritability *τb* = 0.477, *p* < 0.001. Correlation analysis for internalizing behaviors indicated a strong, positive association between anxiety and mood *τb* = 0.534, *p* < 0.001.

#### Preoccupation with getting sick

Caregivers were asked about their child’s preoccupation with getting sick (“The person with ASD has shown an increased preoccupation with getting sick”). Responses were on a 5-point Likert scale and ranged from “Much less than before” to “Much more than before.”

## Results

### Country differences by hospital beds, COVID-19 deaths, and OEI

Our first hypothesis was supported. Results indicated statistically significant differences in preoccupation with getting sick and dysregulated behaviors between Mexico, the country with the highest COVID-19 mortality rate, and the Dominican Republic, the country with the lowest mortality rate (Table 3). Compared to the Dominican Republic, Mexico had significantly higher dysregulated internalizing behaviors, *t*(292) = 3.34, *p* = 0.001, *d* = 0.68, and preoccupation with getting sick, *t*(295) = 2.13, *p* = 0.034, *d* = 0.27.

Furthermore, it was expected that autistic individuals residing in Venezuela, the country with the lowest hospital beds *per capita*, would experience increased preoccupation with getting sick and dysregulated internalizing behaviors when compared to Argentina, the country with the highest hospital beds. Results suggested there were no statistically significant differences between Venezuela and Argentina in terms of dysregulated behaviors (*p* > 0.05) nor in preoccupation with getting sick (*p* > 0.05).

It was also hypothesized that those residing in Brazil, the country with the lowest OEI, would show a greater preoccupation with getting sick and increased dysregulated internalizing behaviors than autistic individuals living in Chile, which had the highest OEI. Compared to Chile, Brazil had significantly higher dysregulated internalizing behaviors, *t*(399) = 3.20, *p* = 0.001, *d* = 0.56, and preoccupation with getting sick, *t*(405) = 3.49, *p* = 0.001, *d* = 0.36.

### Crowded conditions and dysregulated overt behaviors

To determine if contextual factors predicted dysregulated overt behaviors, a linear regression was conducted. Results (see Table 4) indicated that the model explained 15% (*R*^2^ = 0.15) of the variance and that it was significant, *F*(4, 1,702) = 76.22, *p* < 0.01. It was found that worst coexistence significantly predicted dysregulated overt behaviors (*β* = 1.9, *p* < 0.001).

### Screen time use and psychological concerns

A second linear regression was conducted to determine if increased screen time predicted worsening of sleep patterns, increased anxiety, and worsening of mood. Screen time changes did not significantly predict increased dysregulation in sleep patterns, *F*(1, 1,726) = 0.00, *p* > 0.05, or in mood, *F*(1, 1,701) = 3.17, *p* > 0.05, but it significantly predicted increased anxiety, (*F*(1, 1,706) = 5.17, *p* < 0.001, *R*^2^ = 0.003; *β* = 0.05, Table 5).

### Autistic individuals characteristics

#### Gender and dysregulated overt and internalizing behaviors

The third and fourth linear regressions conducted attempted to predict differences in dysregulated overt and internalizing behaviors between both genders of autistic individuals whose caregivers participated in the study. Although it was expected that males would exhibit more overt behaviors and females would show increased internalizing behaviors, gender had no significant effect on dysregulated overt or internalizing behaviors (*p* > 0.05; Table 6).

#### Age and preoccupation with getting sick

Lastly, it was hypothesized that older autistic individuals would exhibit an increased preoccupation with getting sick. Results from a linear regression analysis suggested that age of the autistic individual significantly predicted preoccupation with getting sick, *F*(1, 1,742) = 10.69, *p* = 0.001, *R*^2^ = 0.006, with older age significantly predicting preoccupation with getting sick (*β* = 0.05, *p* = 0.001; Table 7).

## Discussion

It was expected that autistic individuals residing in the country showing highest COVID-19 related deaths (Mexico) would exhibit a greater preoccupation with getting sick and dysregulated internalizing behaviors than those residing in the country with the lowest mortality rate (Dominican Republic). Consistent with our hypothesis, our results indicated that autistic individuals in Mexico experienced increased dysregulated internalizing behaviors which could be explained by the toll of Mexico’s high COVID-19 mortality rate. A systematic review by Santomauro et al. reported increased depressive and anxiety disorders in those countries hit harder by the pandemic, and thus increasing the disability adjusted life years (DALYs) a measure for health years lost due to mortality or disability (40). A study by Sideropoulos et al., which included a cross-country perspective concerning anxiety among those with neurodevelopmental concerns and their family members during COVID-19, also reported increased internalizing concerns, particularly anxiety, during the COVID-19 pandemic. In said study, despite pre-existent anxiety of parents and their children being a predictor of increased anxiety during the pandemic, other factors were identified as influencing its increment. For example, both parents and children with neurodevelopmental concerns experienced increased anxiety during the start of the pandemic due to fears of getting sick. Still, as time passed, parents continued experiencing increased anxiety due to their children’s limited opportunities for social contact as more safety measures were being established to decrease contagion (65). For children with neurodevelopmental concerns, those with medical concerns exhibited increased anxiety (65). These results align with results from the present study, which show the impact of the pandemic in terms of deaths predicted internalizing concerns in autistic children as reported by their parents. It is also possible that due to the increased mortality rate, parents prevented social contact, which exacerbated the overall household anxiety due to limited social contact and outside support being received. Autistic individuals residing in Mexico also experienced increased preoccupation with getting sick. It is important to consider possible factors influencing these differences, such as a country’s size and other economic measures. The Dominican Republic has a population of approximately 11 million people and an area of 48,311 km^2^, making it a significantly smaller country than Mexico, which has a population of roughly 128 million individuals and a size of 1.964.375 km^2^. It could be inferred that containment measures were easier to implement in the Dominican Republic, which was able to manage the spread of COVID-19 during the first few months of the pandemic by controlling movement into the country, implementing curfews and mobility restrictions, limiting gatherings, and closing public services which lowered the risk of illness (66–70). Also, in the Dominican Republic, there were subsidies to assist those employees that had their jobs suspended due to the pandemic and economic assistance to those with informal jobs, students, and those most vulnerable to the COVID-19 virus (71–73). These measures further helped ease the burden of COVID-19.

Due to the severity of the pandemic in terms increased burden on health systems (74), the present study assessed infrastructural differences between the two countries that showed stark differences in terms of hospital beds *per capita* (Argentina and Venezuela). Results yielded no statistical differences in preoccupation with getting sick or dysregulated internalizing behaviors between these two countries. These results should be interpreted with caution due to lack of transparency of public health data in Venezuela. Reports have emerged suggesting a much larger pandemic impact than what has been officially documented, suggesting higher infection rates and COVID-19 related deaths (75). Thus, it is possible that individuals in Venezuela were not adequately informed of the actual impact of the pandemic, and as such, decreasing autistic individuals’ concerns. Another possible explanation is the ongoing economic and sociopolitical crisis being experienced in Venezuela in terms of food shortages, poor economy, fragmented infrastructure (e.g., water and power outages) and limited amount of service providers which, due to their immediate severity, could take the lead in terms of focus of preoccupation and concern (76). In addition, this is not the first infectious disease experienced by the country. Outbreaks of preventable infectious diseases such as measles, malaria, and diphtheria have been an issue in Venezuela in the recent past (75). For this reason, it is possible that those residing in Venezuela, who already experience high levels of psychological distress, could have developed distress tolerance (77). Previous research has shown that despite an association between trauma and adverse mental health, those with high levels of distress tolerance experience less adverse mental health outcomes (78).

When contemplating the OEI, a comparison was made between Brazil, which had the lowest health index, and Chile with the highest index in our sample. The analysis showed statistically significant differences in preoccupation with getting sick and dysregulated internalizing behaviors, with these two being higher in Brazil. To understand these results, it is important to consider the impact of COVID-19 in the local context. Brazil had the first confirmed case of COVID-19 in LATAM by February 2020, while Chile did not get a case until March (79). COVID-19 cases quickly increased in Brazil, making it the third-largest country in the world in terms of infection rate (74) and second in deaths, with over 490 thousand (56). The high and quick rates of infection were also met with a poor national response plan (74). For example, websites that provided public data offered by Brazil’s Health Ministry were taken down, limiting information available to the public concerning the epidemiological status of COVID-19 (80). Additionally, Brazil’s federal government downplayed the severity of the pandemic and discouraged the use of safety protocols (74). Furthermore, the rapid increase of severe COVID-19 cases was met with health systems that quickly collapsed, resulting in high death rates (74). Therefore, it is not surprising that Brazilian participants in this study endorsed increased preoccupation with getting sick and dysregulated internalizing behaviors since the COVID-19 pandemic started. When we observe Chile, not only were overall health systems more efficient, but measures that prepared the country to better handle the pandemic were already in place. For instance, in 2018 Chile had already established a disaster management model, which utilized the knowledge gained from Chile’s previous experiences with natural disasters and included guidelines to support healthcare workers, implementation of protocols, and monitoring of systems for mental health practitioners (79). It is important to note that despite the pandemic precipitating the use of other service delivery modalities in many parts of the world, such as Telehealth, which have shown promising results in treating challenging behaviors (81), in LATAM limited infrastructure and equipment proved difficult for the proper implementation of remote healthcare delivery (82). For example, in LATAM internet connection can be slower and technical support less readily available. In addition, LATAM countries might have strict regulations and policies that could impede the utilization of telehealth platforms, or the opposite; countries might lack regulations making service providers reticent to implement said services (82). Thus, it is unsurprising that in countries like Brazil where healthcare was already problematic before the pandemic, Telehealth was not readily available to help ameliorate the increasing challenging behaviors reported by caregivers.

To better understand changes at the micro-level, this study focused on crowded conditions. Housing size, number of family members residing in the household, having outside space, and family coexistence changes were explored as predictors of worsening of dysregulated overt behaviors. In this model, only the worsening of family coexistence significantly predicted changes in these behaviors. These results could be partially related to ASD core characteristics that make it difficult for autistic individuals to adapt when confronted with disruptions to their routines (83, 84). It is possible that factors such as housing size, outside space, and family members living in the house did not impact participants’ routines, but changes in coexistence did. A systematic review by Yilmaz et al. indicated that anxiety and stress among caregivers increased during the pandemic (85). And thus, this study’s results can help dispel autism misconceptions concerning autistic individuals lacking empathy or awareness of other people’s emotions. Despite autistic individuals often being portrayed as “uncaring,” previous research has shown that while some may have difficulties with cognitive empathy (e.g., understanding other people’s perceptions), they do not have difficulties with emotional empathy (e.g., understanding and sharing others emotions (86)).

Our findings showed screen-time did not predict worsening of sleep patterns, which is inconsistent with previous literature. Associations have been identified between excessive screen time and shorter sleep time among young children, proposing that the light from screens influences melatonin levels, circadian rhythms, and rapid eye movement sleep (87). Yet, for autistic individuals, sleep issues are already a common problem, with 50–86% experiencing sleep issues pre-pandemic (38, 39). The lack of significant results about sleep in our study could be attributable to pre- existing sleep difficulties. If autistic individuals in our sample already had sleep issues, a bedtime routine might have already been in place (88), and thus screen time usage would have not disrupted sleeping patterns. However, increased screen time significantly predicted a slight increase in anxiety in our sample, which is consistent with previous literature showing an association between screen time and anxiety (89). More time spent on electronics, combined with lockdowns, social distance protocols, and changes in treatment modalities could have significantly impacted autistic individuals, and thus explain the increased anxiety levels.

In addition, our findings indicated that dysregulated behaviors were not predicted by gender. Autism research has offered mixed results when comparing internalizing (e.g., anxiety, mood) and externalizing (e.g., aggression) behaviors among genders. For instance, Margari et al. found no statistical differences among both genders in dysregulated behaviors (90). These results seem inconsistent with other literature indicating that females are more likely to experience internalizing behaviors, whereas males are more likely to exhibit externalizing ones (48). In the present sample, one can deduce that decreased social interactions due to containment protocols and reduced social expectations leveled the field in terms of dysregulated behaviors. It is possible that strategies employed by autistic individuals (learned or developed) to hide autistic characteristics for social acceptance (91, 92), decreased during the pandemic due to increased isolation and limited outside contact, accounting for no gender differences in dysregulated internalizing behaviors.

Lastly, it was hypothesized that older autistic individuals (teenagers and adults) would exhibit a greater preoccupation with getting sick. In our sample, as expected, both older individuals showed greater preoccupation than younger children. A recent study by Schott et al. showed that older autistic individuals were at greater risk of COVID-19 exposure due to having been hospitalized more often than neurotypical individuals and to be more likely to either live in a care facility or receive services in the home (93). Therefore, it is imperative to make older individuals a priority in terms of prevention measures by healthcare and service providers (e.g., mask-wearing, hand washing, etc.) and when planning health treatments (e.g., vaccines) (94).

This study has some limitations. First, it lacked longitudinal data that captured individuals’ experiences at different times of the pandemic. Another limitation is the limited information on autism prevalence rates in the region limited generalization. However, the current sample size allowed for a robust statistical power according to our statistical analyses. A third limitation is the lack of a confirmed autism diagnoses by a specialist, since data collected solely consisted of caregiver reports. However, it is worth noting that this does not steer away from the focus of the study, which is to understand caregivers’ perceptions and autistic individuals’ changes when navigating the start of the COVID-19 pandemic. Fourthly, caregivers required internet access to complete the online survey utilized for the present study. Thus, individuals with limited internet accessibility were not included, limiting the representation of the sample. Lastly, some results showed only small effect sizes. This may be due to the quickly changing and complicated nature of the COVID-19 pandemic (e.g., lockdowns, mask mandates, social distancing, development of vaccinations, etc.), as the influence of variables of interest and others not assessed could show stronger effects at different points in time. Additionally, analyses conducted did not control for intellectual ability, language deficits, and symptom severity of autistic individuals, and thus their influence on the different variables of interest could not be determined.

In summary, the COVID-19 pandemic was an acute stressor that was experienced differently in LATAM according to country of residence, age of the autistic individual and family coexistence. Taken all results together, older autistic individuals residing in Brazil and Mexico fared worse than others in terms of preoccupation with getting sick and dysregulated internalizing behaviors. Previous literature has indicated that when compared to caregivers in other LATAM countries, Brazilian caregivers are more likely to report service barriers, lack of information, frustrations when attempting to access services, and overall helplessness when caring for their autistic child (95). It is possible that these barriers to proper care only worsened during the pandemic, increasing autistic individuals’ preoccupation with getting sick and internalizing behaviors. For those residing in Mexico, the pandemic meant a challenging environment due to not only large rates of mortality, but also because of precarious healthcare systems and shortages of safety and preventive equipment (96, 97).

This project has sociopolitical, clinical, and research implications. Some of the most salient findings of this project point towards a need to further investigate countries’ handling of large environmental changes in terms of policies and safety measures, and their impact on autistic individuals. For example, despite our hypothesis expecting countries with better overall health services and infrastructure, such as Chile, to endorse fewer internalizing issues, caregivers reported increases. More research is needed to assess sociopolitical aspects of the pandemic, especially if implemented policies failed to better meet the needs of autistic individuals and their families. Additionally, despite the absence of gender differences in terms of predicting dysregulated behaviors, societal expectations and their impact on dysregulated behaviors when confronted with life stressors need to be further explored, particularly in relation to parental attitudes and expectations. Finally, when exploring contextual factors such as housing size, number of people living in the house, and outdoor space, only worsening of coexistence predicted internalizing behaviors in autistic children. This knowledge can help conceptualize treatments needed in times of unexpected life stressors among autistic individuals to ensure parental stress and coexistence issues do not prove detrimental to their emotional and psychological wellbeing.

## Data availability statement

The original contributions presented in the study are included in the article/supplementary materials, further inquiries can be directed to the corresponding author.

## Ethics statement

The studies involving humans were approved by University of Texas Rio Grande Valley. The studies were conducted in accordance with the local legislation and institutional requirements. Written informed consent for participation was not required from the participants or the participants’ legal guardians/next of kin in accordance with the national legislation and institutional requirements.

## Author contributions

MM: Conceptualization, Formal analysis, Methodology, Writing – original draft, Writing – review & editing. AR: Writing – review & editing. JH: Writing – review & editing. BV: Writing – review & editing. GG: Data curation, Supervision, Writing – review & editing. CA: Data curation, Writing – review & editing. DV: Conceptualization, Data curation, Writing – review & editing. NB: Writing – review & editing. SC: Writing – review & editing. ARa: Writing – review & editing. ARo: Writing – review & editing. RG: Writing – review & editing. CP: Data curation, Writing – review & editing. GL: Writing – review & editing. CM-N: Data curation, Supervision, Writing – review & editing.
